# Emapalumab in pediatric patients with high-grade cytokine release syndrome associated with CAR T-cell therapy

**DOI:** 10.3389/fimmu.2026.1679175

**Published:** 2026-03-04

**Authors:** Jing Zhang, Wenhua Shi, Jing Yang, Juan Qian, Meng Su, Kang An, Tianyi Wang, Rujun Jia, Chengming Fei, Yanjing Tang, Benshang Li

**Affiliations:** Department of Cell Immunotherapy, Shanghai Children’s Medical Center, Shanghai Jiao Tong University School of Medicine, Shanghai, China

**Keywords:** chimeric antigen receptor T-cell, cytokine release syndrome, efficacy, emapalumab, IFN-γ, safety

## Abstract

**Background:**

Chimeric antigen receptor T (CAR-T) cell therapy significantly improves the prognosis of a variety of hematological malignancies; however, its broader application in clinical practice is hindered by adverse events, particularly cytokine release syndrome (CRS). Moreover, the selection of treatment strategies for patients with high-grade CRS must be meticulously tailored. Emapalumab, a fully human IgG1 monoclonal antibody targeting IFN-γ, has been proposed to have clinical benefit in CRS.

**Methods:**

In this retrospective study, we conducted a comprehensive analysis of clinical and laboratory parameters in 38 pediatric patients who failed low-dose glucocorticoids monotherapy, tocilizumab monotherapy or glucocorticoid-tocilizumab combination therapy, following treatment with investigational CAR-T products.

**Results:**

Emapalumab significantly improved both clinical symptoms and laboratory parameters. The rapid decrease in mean temperature (39.61 vs. 38.38°C, *P* < 0.001) and levels of inflammatory markers including IL-2 (32.35 vs. 11.94 pg/ml, *P* < 0.001), IL-10 (222.29 vs. 86.09 pg/ml, *P* = 0.018), TNF-α (4.17 vs. 2.94 pg/ml, *P* = 0.032), and IFN-γ (21984.11 vs. 674.87 pg/ml, *P* < 0.001) indicated the remarkable scavenging efficacy of emapalumab against cytokine storm following CAR-T therapy. Additionally, both mean CAR-T cell counts (549.95 vs. 8.16 cell/μl, *P* < 0.001) and the ratio of CAR-T to CD3+ (11.3% vs. 36.54%, *P* < 0.001) in peripheral blood increased significantly, demonstrating that the administration of emapalumab didn’t seem to have a significant negative impact on the proliferation of CAR-T cells. The median EFS and OS were both not reached, with an EFS rate of 76.9% (95%CI, 63.8-92.6) and with an OS rate of 80.1% (95% CI, 67.7-94.6) at 6 months. Throughout the treatment course, no direct evidence of emapalumab-related safety risks was observed.

**Conclusion:**

Emapalumab seems to serve as an effective salvage therapy for patients experiencing high-grade CRS with inadequate response to low-dose glucocorticoids and/or tocilizumab following CAR-T therapy. These data supported the use of emapalumab in high-grade CRS as well as provide rationale for future prospective studies.

## Introduction

Chimeric antigen receptor (CAR) T-cell therapy is a promising and transformative tumor immunotherapy that has demonstrated high response rates and long-term remission in patients with relapsed or refractory (r/r) leukemia and lymphoma (1). However, CAR-T cell therapy can result in severe, potentially life-threatening toxicities, among which cytokine release syndrome (CRS) is one of the most prevalent. Typically, it is characterized by fever, headache, hypotension, and dyspnea (2). This is particularly relevant in pediatric patients, whose developing immune system and distinct tumor biology may not only alter the presentation of CRS but also contribute to the pathogenesis of its severe forms. Indeed, pediatric patients are reported to be at a higher risk of developing severe CRS compared with their adult counterparts (3). Severe cases of CRS may manifest as an excessive inflammatory state, accompanied by hyperferritinemia, cytopenias, hypofibrinogenemia, and multiorgan dysfunction. This condition bears similarities to hemophagocytic lymphohistiocytosis (HLH) and has recently been defined as immune effector cell-associated HLH-like syndrome (IEC-HS), typically presenting later than CRS (4, 5). The prevalence of CRS varies across different diseases, ranging from approximately 42% to 100%. The incidence of severe cases (≥ Grade 3) can reach up to 47% (6, 7). This poses significant challenges for the development of CAR-T cell therapy CRS results from on-target effects induced by the binding of CAR-T cells or bispecific T-cell engagers (BiTEs) to their corresponding antigens on the surface of target cells, leading to the subsequent activation of bystander immune and non-immune cells, including monocytes/macrophages, dendritic cells, and endothelial cells (8). Upon activation, these immune cells release significant quantities of cytokines, including interleukin-6 (IL-6), IL-10, interferon-gamma (IFN-γ), and tumor necrosis factor (TNF), culminating in a cytokine storm (8–10). Currently, no unified treatment standard exists for the initial management of CRS. Clinical guidelines only suggest a tiered strategy: tocilizumab may be considered for grade 2 CRS, whereas for grade 3–4 CRS, a combination of glucocorticoids, tocilizumab, and supportive care is often considered. However, despite treatment with tocilizumab and glucocorticoids, a subset of patients fails to respond adequately and may even deteriorate, complicating the management of severe CRS (11). There is a critical need to identify safe and effective second-line therapies to control CRS and ICANS after CAR T-cell therapy. Additional agents are currently being explored, such as siltuximab, clazakizumab, and anakinra, which have demonstrated efficacy in mitigating CRS in certain CAR-T cell therapy recipients with high-grade CRS; however, clinical experience remains limited (12–14).

IFN-γ is a crucial cytokine involved in the pathogenesis of CRS. McNerney et al. (15) proposed that targeting IFN-γ is a potential option for CRS that refractory to IL6 inhibits and is ineffective against glucocorticoids. Consistently, Bailey et al. (16) found blocking IFN-γ in CAR-T cells does not impair their cytotoxicity against hematologic tumor cells and paradoxically enhances their proliferation and reduces macrophage-mediated cytokines and chemokines associated with CRS. Further, Manni et al. (17) demonstrated in a humanized mouse model that blocking IFN-γ with emapalumab mitigates the adverse effects of CAR-T cells while preserving their anti-lymphoma efficacy. Emapalumab is a fully human IgG1 monoclonal antibody targeting IFN-γ that binds to both free and receptor-bound IFN-γ, thereby neutralizing its biological activity. Additionally, emapalumab was the first drug approved for the treatment of primary HLH (18), and its efficacy in secondary HLH has also been demonstrated (19, 20). However, only a few case reports have documented the beneficial effects of emapalumab in treating refractory CRS. Currently, there are no reports of large-scale studies.

This study presents the first retrospective analysis of emapalumab in a relatively large cohort of pediatric patients. We focused on children with r/r leukemia or other malignancies who developed CRS refractory to low-dose glucocorticoids and/or tocilizumab after CAR-T therapy, aiming to evaluate the emapalumab ‘s efficacy and safety and to inform clinical practice.

## Materials and methods

### Study design

This study was a single-center retrospective analysis that collected data from patients with refractory CRS who failed to low-dose glucocorticoids monotherapy, or tocilizumab monotherapy or glucocorticoid-tocilizumab combination therapy, following treatment with investigational CAR-T products at Shanghai Children’s Medical Center between January 2024 and August 2024. The study was conducted in accordance with the principles of the Declaration of Helsinki and was approved by the Shanghai Children’s Medical Center Ethics Committee (SCMCIRB-K2025075-1). Owing to the retrospective nature, informed consent was exempted.

### Patients

Eligible patients met the following criteria: (1) aged **<** 18 year**s**; (2) diagnosed with refractory or relapsed malignant hematologic neoplasms or solid tumors; (3) developed CRS following CAR-T therapy; (4) failed to glucocorticoids and/or tocilizumab treatment; (5) received ≥ 1 infusion of emapalumab as monotherapy for CRS salvage treatment. Patients were excluded if they met any of the following conditions: (1) emapalumab administration was indicated for hemophagocytic lymphohistiocytosis (HLH); (2) developed cytokine storm secondary to severe infection prior to emapalumab treatment; (3) had other systemic diseases that could interfere with the study outcomes.

Refractory CRS defined as the absence of marked symptom improvement or continued exacerbation of CRS following treatment with at least one course of low-dose corticosteroids or a single dose of tocilizumab (21). Based on the ASTCT consensus (22) for corresponding grading and clinical management, and combined by institutional experience, our hospital considers the initiation of glucocorticoids therapy under the following indications: (1) When the patient’s body temperature remains >39.5 °C despite the use of tocilizumab and antipyretics; (2) When hypotension develops and requires norepinephrine at a dose exceeding 0.3 μg/kg/min or the need for two or more vasoactive agents; (3) During CRS, if daily cytokine monitoring shows a key indicator such as IFN-γ exceeding 5000 pg/ml, corticosteroids or emapalumab may be administered.

### Treatment

Prior to the infusion of CAR-T cells, patients underwent lymphodepleting chemotherapy regimens comprising fludarabine, cyclophosphamide or total body irradiation (TBI) or ICE (ifosfamide, carboplatin, and etoposide) therapy. The median occurrence time of CRS was 0 (0-3) days after CAR-T infusion, and the median time to initiation of emapalumab was 5 (1-8) days after CAR-T. No recommended pediatric dosage for CRS is specified in the prescribing information for emapalumab; therefore, the administration regimen was determined based on the clinical experience of our center and relevant literature (15, 23), combined with the advice of clinician and with the consent of parents and their parents or guardians. The median dose was 0.37 mg/kg (0.12-0.82 mg/kg), which was equivalent to a fixed dose of 10 mg. For some older pediatric patients or those with severe CRS manifestations, a dose of 20 mg could be administered based on individual clinical needs. Emapalumab was administered intravenously, with a median of 1 infusion (range: 1–2). All patients treated with emapalumab received preventive anti-infective treatment as per the instructions, including ganciclovir (5 mg/kg, q12h) or acyclovir (10 mg/kg, q8h) for virus prevention, sulfamethoxazole (12.5 mg/kg, bid, three times per week with a four-day break) for pneumocystis carinii pneumonia prevention, and fluconazole (5 mg/kg, qd) for fungal prevention.

### Data collection and definition

Retrospective data collection included demographics, Eastern Cooperative Oncology Group (ECOG) performance status scores, past medical history, and types of CAR-T products. At the same time, comprehensive data were systematically collected on CRS, immune effector cell-associated neurotoxicity syndrome (ICANS), co-infections, CRS-related manifestations and treatment strategies, as well as laboratory test results including blood cell parameters, inflammatory cytokines, infection markers, and biochemical indices. The blood sample collection time was before the infusion of emapalumab (baseline) and on the third day after the start of emapalumab infusion. We primarily analyzed the improvement of laboratory indicators, remission of CRS, overall survival (OS), and Event-free survival (EFS) of patients following emapalumab treatment. Subgroup analysis was stratified based on underlying disease type, CAR-T product type, lymphodepleting chemotherapy regimens, and prior CRS treatment history.

OS is defined as the time from the date of emapalumab treatment to the date of cancer-related death or the date of last follow-up for patients who were alive and censored. EFS is defined as the time from treatment with emapalumab to the first recurrence/death or last follow-up. CRS and ICANS grades at the time of emapalumab administration were noted through retrospective chart review according to the American Society for Transplantation and Cellular Therapy (ASTCT) Consensus Grading (22). Notably, Immune Effector Cell-associated Encephalopathy (ICE) score was applied to patients over 12 years old, while the CAPD score was employed for patients ≤12 years old.

### Statistical analysis

Statistical analyses were performed using R Studio 4.3.1. Patient characteristics were summarized by median and range for continuous variables, as well as frequency and percentage for categorical variables. Comparisons of indicators before and after medication administration were carried out using either the paired t-test or the Wilcoxon signed-rank test. OS and EFS rates were estimated employing the Kaplan-Meier methodology. *P* < 0.05 was considered statistically significant.

## Results

### Patients’ characteristics

A total of 38 patients diagnosed with refractory CRS following CAR-T therapy were included in this study, including 15 females and 23 males. The median age of the patients was 9 years (range: 2-16). The predominant disease type was B-cell acute lymphoblastic leukemia (B-ALL), identified in 32 patients (84%), and 8 patients (21%) had undergone allogeneic hematopoietic stem cell transplantation (HSCT). Prior to CAR T-cell infusion, patients received a lymphodepleting chemotherapy regimen or ICE load reduction therapy. The median percentage of evaluable minimal residual disease in the bone marrow of 33 patients before CAR-T cell infusion was 22% (range: 1.04% to 92.27%) and 5 patients had negative MRD in the bone marrow. A large proportion of patients (28 patients, 74%) received CD19/CD22/CD72 CAR-T. Thirty-seven patients (97%) were classified as high-grade CRS (≥ Grade 3), and 24 patients experienced ICANS following CAR-T cell therapy. Additionally, organ toxicities were observed including liver dysfunction in 28 patients, renal dysfunction in 9 patients, pulmonary dysfunction in 24 patients, cardiac dysfunction in 26 patients, gastrointestinal discomfort in 12 patients, and coagulation abnormalities in 30 patients. Twenty-one patients developed infections following CAR-T cell infusion, including 10 cases of viral infections, 9 cases of bacterial infections, and 8 cases of fungal infections. For the first-line treatment of CRS, a total of 31 patients received glucocorticoids with a cumulative dose of 10.00 mg (range: 3.00-78.75 mg), while 33 patients received tocilizumab with a cumulative dose of 480 mg (range: 160–1280 mg). Additionally, 37 patients received vasopressors as inotropic support. The detailed characteristics of the patients were shown in **Table 1**.

**Table 1:** Baseline characteristics of patients.

Characteristics	Total (N = 38)
Age, median (range)	9 (2, 16)
Gender, female/male	15/23
Weight, median (range)	27.25 (13.50, 80.55)
ECOG score, median (range)	1 (1, 2)
Underlying disease, N (%)	
B-ALL	32 (84.21)
Burkitt’s lymphoma	3 (7.89)
T-ALL	1 (2.63)
AML	1 (2.63)
Neuroblastoma	1 (2.63)
HSCT history, N (%)	8 (21.05)
Therapy prior to CAR-T, N (%)	
Lymphodepleting chemotherapy,	
Fludarabine, Cyclophosphamide	34 (89.47)
Fludarabine, Cyclophosphamide, TBI	3 (7.89)
ICE regimen^‡^	1 (2.63)
CAR-T cell targets, N (%)	
CD19/CD22/CD72	28 (73.68)
CD19/CD22	4 (10.53)
CD19/CD22/CD20	2 (5.26)
CD22/CD72	1 (2.63)
CD7/CD99	1 (2.63)
CD33/CLL1	1 (2.63)
GD2	1 (2.63)
% of MRD in the bone marrow before CAR-T cell infusion, median (range)	21.78 (0, 92.27)
CAR-T cell infusion dose, ×10^6^ cells/kg, median (range)	6.00 (2.19, 8.62)
Grade of CRS, N (%)	
Grade 1^*^	1 (2.63)
Grade 3	13 (34.21)
Grade 4	24 (63.16)
Grade of ICANS, N (%)	
Grade 0	14 (36.84)
Grade 1	15 (39.47)
Grade 3	9 (23.68)
Organ involvement, N (%)	
Liver dysfunction	28 (73.68)
Renal dysfunction	9 (23.68)
Pulmonary dysfunction	24 (63.16)
Cardiac dysfunction	26 (68.42)
Gastrointestinal discomfort	12 (31.58)
Coagulation abnormalities	30 (78.95)
Infection, N (%)	
Virus	10 (26.32)
Bacteria	9 (23.68)
Fungus	8 (21.05)
Medications for treatment, N (%)	
First-line treatment for CRS	
Glucocorticoid only^‡‡^	5 (13.2)
Tocilizumab only	7 (18.4)
Glucocorticoid+ Tocilizumab	26 (68.4)
Inotropic support	
Norepinephrine only	12 (31.6)
Norepinephrine + Terlipressin	20 (52.6)
Norepinephrine + Epinephrine+ Terlipressin	5 (13.2)

^‡^One patient with ALL had severe extramedullary involvement and used the ICE regimen to reduce disease burden before the infusion of CAR-T cells. Subsequently, the patient’s lymphocytes had dropped to zero, so no additional lymphodepleting chemotherapy was administered. ^*^The patient developed severe localized CRS manifestations, characterized by orbital tumor swelling; ophthalmological evaluation indicated a risk of blindness, and the patient’s serum interferon-γ (IFN-γ) level exceeded 5000 pg/ml. ^‡‡^The dose of glucocorticoid was the equivalent dose of dexamethasone. B-ALL, B-cell acute lymphoblastic leukemia; T-ALL, T-cell acute lymphoblastic leukemia; AML, acute myeloid leukemia; ECOG, Eastern Cooperative Oncology Group; HSCT, allogeneic hematopoietic stem-cell transplantation; MRD, minimal residual disease; TBI, total body irradiation; ICE, ifosfamide, carboplatin and etoposide; CRS, cytokine release syndrome; ICANS, immune effector cell-associated neurotoxicity syndrome.

### Clinical outcomes

#### Changes in body temperature and laboratory parameters after emapalumab treatment

**Figure 1:**
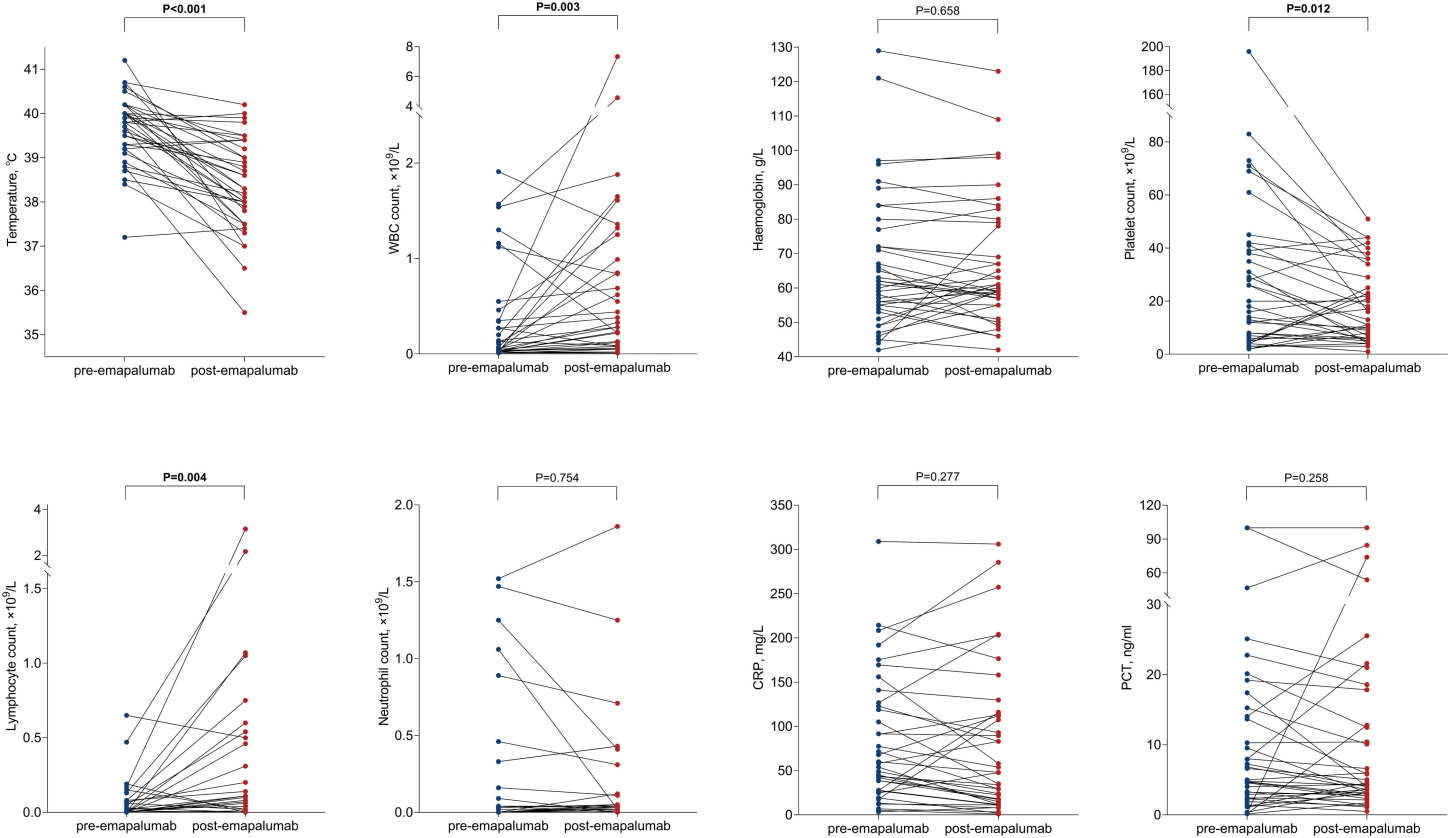
Changes in relevant parameters before and after emapalumab treatment. WBC, white blood cell; CRP, C reactive protein; PCT, procalcitonin.

**Figure 2:**
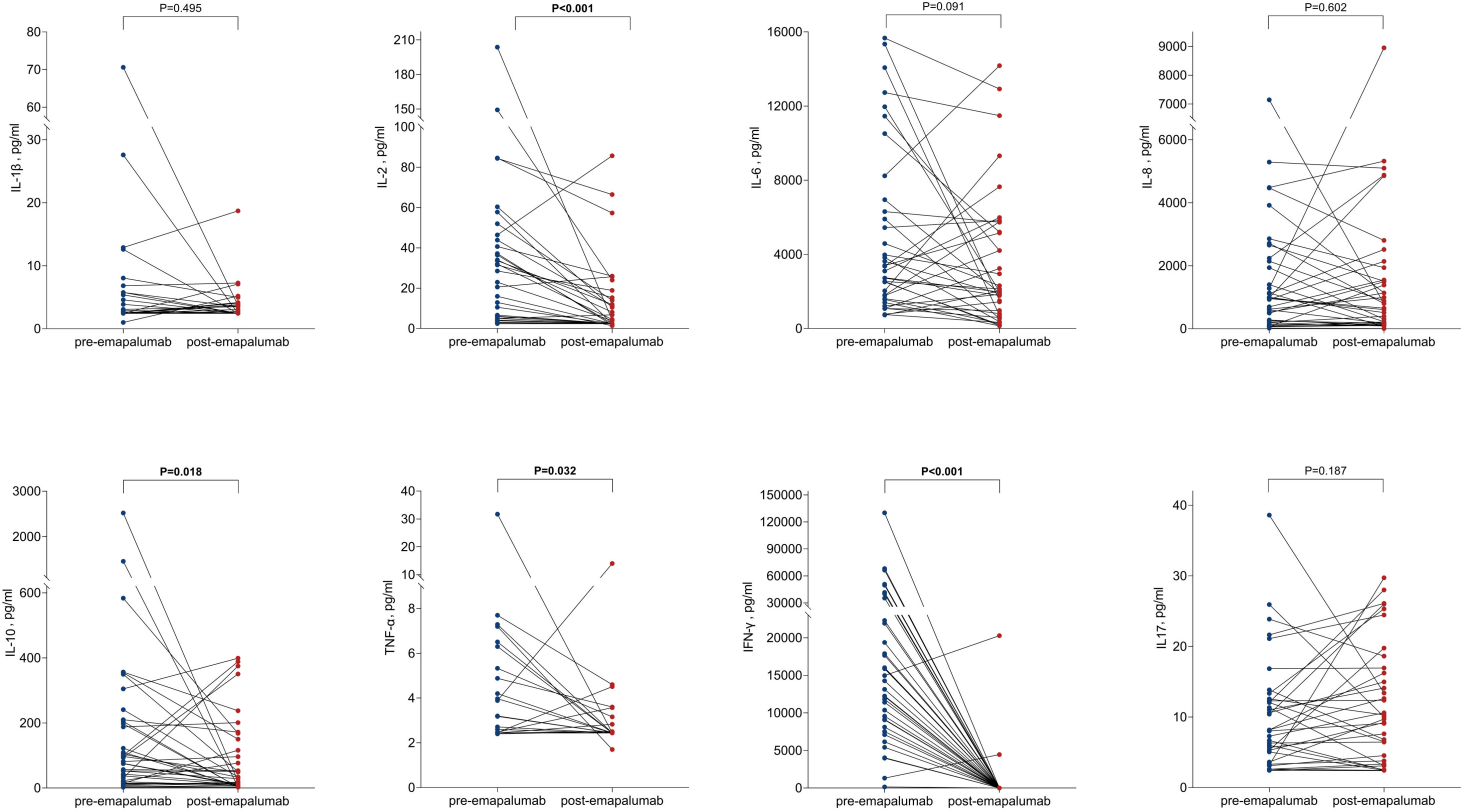
Analysis of inflammatory cytokines before and after emapalumab application.

We collected data on body temperature and laboratory parameters of patients both before and 3 days after treatment with emapalumab (**Figure 1**). The mean body temperature of patients prior to treatment with emapalumab was 39.61 **±** 0.78°C, while the body temperature of patients after treatment significantly decreased to 38.38 ± 1.03°C (*P* < 0.001), indicating that emapalumab was effective in relieving fever in patients with refractory CRS. In hematologic evaluation, we found that emapalumab treatment significantly increased white blood cell (WBC) counts (0.32 vs. 0.76×10^9^/L, *P* = 0.003) and lymphocyte counts (0.06 vs. 0.30×10^9^/L, *P* = 0.004), while markedly reducing platelet counts (29.68 vs. 18.74×10^9^/L, *P* = 0.012) (**Figure 1**, **Table 2**). In addition, cytokine level assessments indicated that emapalumab significantly reduced the IL-2 (32.35 vs. 11.94 pg/ml, *P* < 0.001), IL-10 (222.29 vs. 86.09 pg/ml, *P* = 0.018), TNF-α (4.17 vs. 2.94 pg/ml, *P* = 0.032), and IFN-γ (21984.11 vs. 674.87 pg/ml, *P* < 0.001). Although no statistically significant differences were observed in IL-1β (6.14 vs. 3.56 pg/ml), IL-6 (4877.58 vs. 3507.07 pg/ml) and IL-8 (1467.65 vs. 1470.07 pg/ml), a downward trend was noted in these cytokines (all *P* > 0.05) (**Figure 2**, **Table 2**). This suggested that emapalumab can effectively inhibit cytokine storms. Notably, it appears that emapalumab administration did not impose an additional burden on pre-existing severe infections, severe myocardial injury, or hepatic injury, which was supported by the relatively stable levels of C-reactive protein (CRP), procalcitonin (PCT), B-type natriuretic peptide (BNP) and creatinine (**Figure 1**, **Table 2**).

**Table 2:** Changes in clinical and laboratory parameters after emapalumab treatment- Overall.

	Baseline	Post-treatment	*P* value
Body temperature, °C	39.61 ± 0.78	38.38 ± 1.03	<0.001
White blood cell, 10^9^/L	0.32 ± 0.52	0.76 ± 1.39	0.003
Hemoglobin, g/L	66.97 ± 20.24	67.16 ± 18.47	0.658
Platelet, 10^9^/L	29.68 ± 35.67	18.74 ± 14.24	0.012
Lymphocyte, 10^9^/L	0.06 ± 0.13	0.30 ± 0.64	0.004
Neutrophils, 10^9^/L	0.20 ± 0.43	0.16 ± 0.38	0.754
Interleukin-1β, pg/ml	6.14 ± 11.87	3.56 ± 2.84	0.495
Interleukin-2, pg/ml	32.35 ± 42.28	11.94 ± 19.10	<0.001
Interleukin-6, pg/ml	4877.58 ± 4478.32	3507.07 ± 3641.64	0.091
Interleukin-8, pg/ml	1467.65 ± 1697.08	1470.07 ± 1975.43	0.602
Interleukin-10, pg/ml	222.29 ± 464.78	86.09 ± 120.28	0.018
Interleukin-17, pg/ml	9.94 ± 7.96	11.35 ± 8.46	0.187
TNF-α, pg/ml	4.17 ± 4.93	2.94 ± 1.95	0.032
Interferon-γ, pg/ml	21984.11 ± 24968.68	674.87 ± 3393.05	<0.001
BNP, pg/ml	2342.17± 2464.49	3773.06± 6695.47	0.800
ALT, U/L	88.89 ± 89.50	88.95 ± 76.15	0.574
Creatinine, μmol/L	40.16± 21.09	45.24± 30.37	0.771
C-reactive protein, mg/L	81.49 ± 71.42	79.43 ± 83.15	0.277
Procalcitonin, ng/ml	13.20 ± 22.69	14.39 ± 23.68	0.258

TNF-α, tumor necrosis factor α; BNP, B-type natriuretic peptide; ALT, alanine aminotransferase. Data are presented as Mean ± SD.

Due to the uneven sample sizes across subgroups in this retrospective study, to ensure analytical stability, we focused on analyzing the subgroup with the largest number of cases within each stratification dimension. The analysis showed that among patients with the most common underlying disease (BALL), those receiving the most frequently used CAR-T product (CD19/CD22/CD72), those undergoing the most common lymphodepleting chemotherapy regimen (fludarabine +cyclophosphamide), and those with the most prevalent prior CRS treatment (glucocorticoid+ focilizumab), the dynamic temperature change curves and the evolving trends of key laboratory parameters were largely consistent with the patterns observed in the overall population. These findings indicate that the primary results of this study are generally applicable across the core patient subgroups that constitute most of the cohort, further supporting the robustness of the overall conclusions (**Tables 3**, **4**).

**Table 3:** Changes in clinical and laboratory parameters after emapalumab treatment-B-ALL and CD19/CD22/CD72.

	Baseline	Post-treatment	*P* value
B-ALL			
Body temperature, °C	39.63 ± 0.78	38.36 ± 1.08	<0.001
White blood cell, 10^9^/L	0.32 ± 0.53	0.83 ± 1.49	0.014
Hemoglobin, g/L	68.06 ± 21.28	68.69 ± 18.86	0.677
Platelet, 10^9^/L	30.19 ± 37.90	19.19 ± 14.39	0.047
Lymphocyte, 10^9^/L	0.06 ± 0.14	0.34 ± 0.69	0.017
Neutrophils, 10^9^/L	0.20 ± 0.42	0.17 ± 0.41	0.305
Interleukin-1β, pg/ml	6.86 ± 12.88	3.76 ± 3.07	0.622
Interleukin-2, pg/ml	34.92± 44.85	13.21 ± 20.59	0.001
Interleukin-6, pg/ml	4967.08 ± 4688.02	3629.09 ± 3882.29	0.136
Interleukin-8, pg/ml	1584.69 ± 1792.29	1612.16 ± 2117.66	0.748
Interleukin-10, pg/ml	256.56 ± 500.90	95.31 ± 125.54	0.027
Interleukin-17, pg/ml	9.74 ± 8.55	11.54 ± 9.11	0.568
TNF-α, pg/ml	3.56 ± 1.73	3.04 ± 2.12	0.064
Interferon-γ, pg/ml	23958.55 ± 26759.97	804.20 ± 3702.54	<0.001
BNP, pg/ml	2697.60 ± 2554.29	4290.43 ± 7197.06	0.836
ALT, U/L	90.61 ± 85.33	92.00 ± 75.06	0.531
Creatinine, μmol/L	40.50 ± 19.93	46.39 ± 31.51	0.918
C-reactive protein, mg/l	77.95 ± 72.69	70.24 ± 78.47	0.292
Procalcitonin, 10^9^/L	12.04 ± 18.79	14.23 ± 24.56	0.752
CD19/CD22/CD72			
Body temperature, °C	39.61 ± 0.77	38.40 ± 1.11	<0.001
White blood cell, 10^9^/L	0.29 ± 0.47	0.87 ± 1.58	0.020
Hemoglobin, g/L	67.86 ± 22.02	68.93 ± 19.13	0.629
Platelet, 10^9^/L	25.64 ± 23.94	18.36 ± 14.01	0.512
Lymphocyte, 10^9^/L	0.06 ± 0.15	0.37 ± 0.73	0.022
Neutrophils, 10^9^/L	0.17 ± 0.38	0.15 ± 0.38	0.392
Interleukin-1β, pg/ml	7.46 ± 13.73	3.96 ± 3.25	0.725
Interleukin-2, pg/ml	31.99 ± 42.15	13.45 ± 21.84	0.003
Interleukin-6, pg/ml	5028.57 ± 4750.52	3783.14 ± 4114.22	0.138
Interleukin-8, pg/ml	1656.90 ± 1874.60	1791.10 ± 2214.79	0.986
Interleukin-10, pg/ml	284.50 ± 532.13	104.70 ± 131.97	0.058
Interleukin-17, pg/ml	8.96 ± 8.19	11.54 ± 9.16	0.401
TNF-α, pg/ml	3.61 ± 1.81	3.09 ± 2.27	0.098
Interferon-γ, pg/ml	25836.17 ± 28235.15	922.55 ± 3962.97	<0.001
BNP, pg/ml	2474.85 ± 2415.12	4573.96 ± 7692.20	0.701
ALT, U/L	90.15 ± 87.73	95.41 ± 79.40	0.505
Creatinine, μmol/L	40.79 ± 20.62	47.74 ± 33.12	0.913
C-reactive protein, mg/L	72.53 ± 72.24	66.64 ± 80.75	0.298
Procalcitonin, ng/ml	12.22 ± 19.92	12.67 ± 23.52	0.572

B-ALL, B-cell acute lymphoblastic leukemia; TNF-α, tumor necrosis factor α; BNP, B-type natriuretic peptide; ALT, alanine aminotransferase. Data are presented as Mean ± SD.

**Table 4:** Changes in clinical and laboratory parameters after emapalumab treatment-B-ALL and CD19/CD22/CD72.

	Baseline	Post-treatment	*P* value
Fludarabine + Cyclophosphamide			
Body temperature, °C	39.64 ± 0.79	38.32 ± 1.04	<0.001
White blood cell, 10^9^/L	0.32 ± 0.52	0.80 ± 1.46	0.020
Hemoglobin, g/L	67.50 ± 21.02	67.53 ± 19.14	0.764
Platelet, 10^9^/L	31.97 ± 37.06	19.79 ± 14.61	0.357
Lymphocyte, 10^9^/L	0.05 ± 0.09	0.32 ± 0.67	0.048
Neutrophils, 10^9^/L	0.23 ± 0.45	0.17 ± 0.40	0.602
Interleukin-1β, pg/ml	6.59 ± 12.52	3.64 ± 3.00	0.517
Interleukin-2, pg/ml	32.95 ± 44.45	10.00 ± 15.18	<0.001
Interleukin-6, pg/ml	4937.56 ± 4665.99	3226.76 ± 3288.57	0.079
Interleukin-8, pg/ml	1511.65 ± 1776.88	1414.69 ± 1982.31	0.799
Interleukin-10, pg/ml	237.92 ± 488.67	81.56 ± 113.22	0.010
Interleukin-17, pg/ml	10.54 ± 8.20	11.63 ± 8.76	0.710
TNF-α, pg/ml	4.31 ± 5.20	2.91 ± 2.04	0.030
Interferon-γ, pg/ml	23968.74 ± 25704.99	620.92 ± 3529.74	<0.001
BNP, pg/ml	2489.79 ± 2521.39	3816.44 ± 6977.57	0.990
ALT, U/L	89.85 ± 93.72	88.67 ± 77.84	0.547
Creatinine, μmol/L	40.62 ± 22.27	46.06 ± 31.64	0.711
C-reactive protein, mg/L	76.97 ± 67.00	74.91 ± 79.10	0.429
Procalcitonin, ng/ml	13.95 ± 23.86	15.12 ± 24.73	0.840
Glucocorticoid + Tocilizumab			
Body temperature, °C	39.45 ± 0.85	38.25 ± 1.10	<0.001
White blood cell, 10^9^/L	0.20 ± 0.41	0.84 ± 1.64	0.018
Hemoglobin, g/L	64.69 ± 21.16	66.35 ± 18.17	0.394
Platelet, 10^9^/L	16.73 ± 15.27	14.27 ± 11.39	0.855
Lymphocyte, 10^9^/L	0.06 ± 0.16	0.40 ± 0.75	0.011
Neutrophils, 10^9^/L	0.08 ± 0.26	0.05 ± 0.14	0.350
Interleukin-1β, pg/ml	7.07 ± 14.26	3.03 ± 1.05	0.578
Interleukin-2, pg/ml	33.44 ± 47.28	10.44 ± 17.54	0.002
Interleukin-6, pg/ml	5834.74 ± 4982.14	4043.79 ± 4174.51	0.104
Interleukin-8, pg/ml	1909.75 ± 1846.29	1868.11 ± 2265.04	0.564
Interleukin-10, pg/ml	262.38 ± 556.81	90.44 ± 137.24	0.028
Interleukin-17, pg/ml	9.96 ± 8.53	9.93 ± 7.91	>0.999
TNF-α, pg/ml	4.76 ± 5.91	2.67 ± 0.67	0.003
Interferon-γ, pg/ml	24804.59 ± 28028.54	995.22 ± 4115.77	<0.001
BNP, pg/ml	2668.92 ± 2673.10	3487.04 ± 4940.36	0.861
ALT, U/L	85.69 ± 80.76	88.73 ± 68.71	0.589
Creatinine, μmol/L	41.65 ± 22.90	46.46 ± 29.00	0.498
C-reactive protein, mg/L	84.01 ± 79.84	72.33 ± 93.21	0.142
Procalcitonin, ng/ml	14.46 ± 25.90	11.52 ± 21.16	0.390

TNF-α, tumor necrosis factor α; BNP, B-type natriuretic peptide; ALT, alanine aminotransferase. Data are presented as Mean ± SD.

#### Prognosis after emapalumab treatment

**Figure 3:**
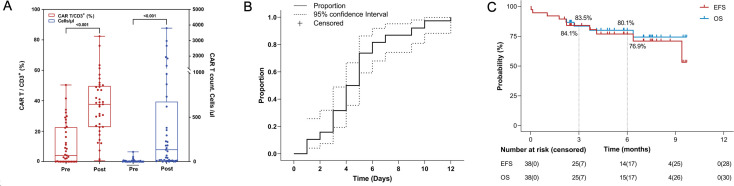
Response and long-term survival of patients following CAR-T therapy. **(A)** CAR-T cell counts in peripheral blood before and after emapalumab treatment; **(B)** The cumulative curve from the start of emapalumab treatment to the end of CRS; **(C)** Kaplan-Meier curve for EFS and OS. CAR-T, Chimeric Antigen Receptor T-Cell; CRS, cytokine release syndrome; EFS, Event-free survival; OS, overall survival.

After emapalumab treatment, both mean CAR-T cell counts (549.95 vs. 8.16 cell/μl, *P* < 0.001) and the ratio of CAR-T to CD3^+^ (11.3% vs. 36.54%, *P* < 0.001) in peripheral blood increased significantly (**Figure 3A**), demonstrating that the administration of emapalumab did not appear to compromise short-term CAR-T expansion. By day 12, the proportion of patients who achieved CRS symptom resolution approached 1.0, indicating that emapalumab was able to effectively control CRS within a relatively short period in most patients (**Figure 3B**). After the CAR-T therapy, a total of 35 patients achieved negative MRD. The initial manifestation in 1 patient was extramedullary masses without involvement of the bone marrow and 1 patient was diagnosed with neuroblastoma.

The median follow-up duration for the patients was 4.83 months (range: 0.03-9.70 months). The median EFS was not reached, with an EFS rate of 84% (95%CI, 73.1-96.6) at 3 months and 77% (95%CI, 63.8-92.6) at 6 months. The median OS was not reached, with an OS rate of 83.5% (95% CI, 72.3-96.6) at 3 months and 80% (95% CI, 67.7-94.6) at 6 months (**Figure 3C**).

A total of 8 patients ultimately succumbed to death. Specifically, 2 deaths were attributed to disease recurrence or progression, 4 were caused by severe infections, and the remaining 2 resulted from CRS complicated with septic shock. Notably, before the administration of emapalumab, 7 patients had already experienced infections symptoms, including *Enterococcal*, *Trichosporon asahii*, *Candida tropicalis*, adenovirus, parainfluenza virus, rhinovirus and HHV6, etc. (**Table 5**). Although no definite pathogen was detected in 3 patients, the suspected infections all occurred on the same day of CAR-T infusion, which was consistent with the onset time of fever. Therefore, a causal relationship between emapalumab and infection risk could not be established. In addition, 2 patients who died of CRS combined septic shock had an extremely high disease burden prior to CAR-T infusion. Importantly, throughout the treatment course, no direct evidence of emapalumab-related safety risks was observed.

**Table 5:** Death analysis.

Death (N = 8)	Reason for death	Previous infection with pathogens
Patient 1	Disease recurrence	*Enterococcus*
Patient 2	Progressive disease	No
Patient 3	CRS combined with septic shock	Unknown
Patient 4	CRS combined with septic shock	Unknown
Patient 5	Fungemia	*Trichosporon asahii*
Patient 6	Encephalitis	Adenovirus, Parainfluenza virus, Rhinovirus, HHV6
Patient 7	Encephalitis	*Candida tropicalis*, HHV-6
Patient 8	Serious infection	Unknown

## Discussion

Although CAR-T cell therapy has revolutionized the treatment of hematological malignancies, its associated severe toxicity may impair its efficacy and even be life-threatening, which makes CAR-T cell therapy more complicated. As the most common complication, CRS reflects antigen non-specific toxicity and can induce systemic inflammatory responses (2, 24). Effective control and management of CRS are crucial for optimizing the efficacy of CAR-T therapy and addressing an important need for patients. Here, we reported the data of 38 patients with life-threatening CRS after CAR-T cell who benefited from emapalumab treatment. As far as we know, this study represents the largest sample of pediatric retrospective analysis documented in the literature to date.

Following the development of CRS, patients enrolled in this study were initially administered low-dose glucocorticoids and/or tocilizumab, yet their CRS-related symptoms showed no marked amelioration and even progressed to a more severe state. Therefore, we implemented emapalumab as salvage treatment to address life-threatening situations. Fortunately, three days after treatment with emapalumab, the body temperature of patients significantly decreased, which is a direct manifestation of the alleviation of inflammatory response, indicating its ability to effectively control the systemic inflammatory response of CRS. Furthermore, the reduction in the levels of inflammatory factors after treatment further confirmed the anti-inflammatory effect of emapalumab. Meanwhile, we also observed changes in blood cells. During the peak period of CRS, CAR-T cells migrate from the bone marrow to the peripheral blood, resulting in a significant increase in WBC and lymphocytes in patients. The possible reason for the decrease in platelets is that the patient’s bone marrow MRD load is relatively high before infusion of CAR-T cells, and CAR-T cells produce bone marrow suppression. Since emapalumab is precisely applied during the peak period of CRS, changes in WBC, lymphocytes and platelets during the same period can be observed (25, 26). Notably, it appears that emapalumab administration did not impose an additional burden on pre-existing severe infections, severe myocardial injury, or hepatic injury, which was supported by the relatively stable levels of CRP, PCT, BNP and creatinine. Given that the acute, systemic inflammation induced by CRS can exert significant negative effects on host metabolism, the effective control of CRS during treatment represents a significant clinical benefit in mitigating the deterioration of systemic parameters. Despite the limitation of uneven subgroup sample sizes inherent to this retrospective study, the primary patterns were consistently replicated in the largest patient subgroups within each key classification. This suggested that our core findings are, at a minimum, robust and applicable to the most common patient profiles encountered in clinical practice, thereby providing a rationale for future prospective studies to focus on these predominant populations. Overall, emapalumab serves as an effective salvage therapy for patients with CRS.

More importantly, IFN-γ is a cytokine that plays a role in the anti-tumor activity of CAR-T cells. Previous study has shown that anti-IFN-γ treatment can reduce immune-related adverse reactions without affecting the success of the treatment, and neutralizing IFN-γ does not affect the clearance of lymphoma cells by CAR-T cells (17). After receiving emapalumab, the counts and percentages of lymphocytes and CAR-T cells in peripheral blood increased, suggesting the expansion of CAR-T cells in the body. Moreover, after a longer follow-up, these patients experienced remission of the underlying blood cancer and achieved negative MRD in bone marrow, indicating that the efficacy of anti-IFN-γ did not affect the therapeutic outcomes of CAR-T therapy on malignant tumors. However, we should note that the relevance of IFN-γ for efficacy greatly varies between B cell malignancies versus myeloid or solid tumors. In B-cell malignancies, the role of IFN-γ is mainly related to the inflammatory response rather than directly involved in the anti-tumor mechanism of CAR-T cells. However, in myeloid tumors or tumor solids, the role of IFN-γ is more complex. IFN-γ not only participates in the inflammatory response, but may also directly regulate the anti-tumor activity of CAR-T cells (27).

Next, we reviewed the reports on the effects of glucocorticoids and tocilizumab on the proliferation of CAR-T cells. Data from Poiret et al. indicate that glucocorticoid can inhibit the proliferation of CAR-T cells, especially at high concentrations, which may affect the efficacy of CAR-T cell therapy (28). However, tocilizumab has been reported to have no significant effect on CAR-T cell proliferation and does not affect the long-term efficacy of CAR-T cells (29). Therefore, when administering drugs, clinicians should balance the relationship between the drugs in alleviating CRS and maintaining the anti-tumor activity of CAR-T cells. However, it should be clarified that a direct comparison among emapalumab, glucocorticoids and tocilizumab remain not feasible to date. The core reason lies in the distinct temporal differences in clinical treatment sequences across the three cohorts: all patients administered emapalumab were those with refractory severe CRS who had failed first-line therapies, including glucocorticoids monotherapy, tocilizumab monotherapy, or their combination. Thus, emapalumab was used as a second-line salvage therapy in this study. Accordingly, designing a control group of “emapalumab-only treatment without prior first-line agents” was not permissible under current clinical practice guidelines and ethical frameworks, as all CRS patients are required to receive standard first-line treatment upfront. Perhaps future prospective studies, stratified by disease severity at an early stage, could explore predefined head-to-head comparisons of different therapeutic strategies on CAR-T cell expansion to yield more definitive conclusions.

During the treatment of CRS, the rapid changes in the immune system may make patients more vulnerable to infection. Theoretically, emapalumab, an agent targeting IFN-γ, may attenuate the core immune defense pathways against pathogens while suppressing cytokine storm, thus carrying the risk of inducing excessive immune suppression, elevating susceptibility to opportunistic infections, and resulting in fatal outcomes in cases of severe infection. However, among the 8 patients who died in this study, 7 patients had developed infections before the administration of emapalumab. For the 6 patients who died of severe infection or disease progression in this study, these two causes of death are precisely typical outcomes among patients with cancer and those undergoing intensive immunotherapy, and their occurrence is closely associated with the malignancy of the patients’ underlying disease and treatment-related immune perturbations. The remaining 2 patients had an extremely high disease burden prior to CAR-T infusion and developed grade 5 CRS after infusion; despite salvage therapy of emapalumab, their fulminant pathophysiological processes could not be reversed. This underscores the life-threatening nature of the disease targeted by this study. Therefore, there was currently no evidence suggesting that the patient’s death was caused by emapalumab. However, this reminds us that in clinical practice, it is necessary to closely monitor the symptoms of patients, timely identify CRS and infection, and take corresponding treatment and preventive measures. Meanwhile, the mortality rate observed in this study essentially reflects the natural history of refractory CRS and malignancy itself; this means that when evaluating the benefit-risk profile of emapalumab, the inherently high lethality of the disease must be incorporated as a core context. The monitoring of the long-term safety and potential side effects of emapalumab is also an important direction for future research.

Currently, the approved indications for emapalumab are primarily for primary HLH and specific secondary HLH (such as Still’s disease-associated HLH/MAS). Its use for CAR-T-related CRS remains exploratory, with no established standard dosage. Although both are cytokine storm syndromes, their core driving pathological mechanisms differ. HLH is primarily driven by the persistent overproduction of IFN-γ, thus standard treatment requires higher doses of emapalumab to achieve complete blockade. In contrast, the core cytokine in CAR-T-associated CRS is IL-6, and IFN-γ levels are typically significantly lower than those seen in classic HLH. Consistent with existing consensus, patients in this study initially received glucocorticoids, tocilizumab, or their combination, but all treatments failed. Retrospective analysis the data showed that several pediatric patients had rapidly progressive, severe CRS, with a more prominent elevation in IFN-γ than IL-6 levels, justifying emapalumab (an IFN-γ-targeted agent) as salvage therapy. Notably, the emapalumab dosage was optimized for CRS (not directly adopted from HLH regimens), based on the drug’s prescribing information, relevant literature, and our center’s clinical experience. Our prior experience indicated that low-dose emapalumab could effectively reduce IFN-γ levels, control CRS, and alleviate the economic burden due to its high cost. Encouragingly, emapalumab successfully reversed CRS, providing valuable evidence for refractory CAR-T-related CRS management. However, most patients received only one low-dose infusion of emapalumab. A more appropriate dosage and dosing schedule need to be determined through pharmacokinetic studies, as many CRS patients have very high levels of IFN-γ and may require a higher dose of emapalumab (30).

Although we have initially confirmed the benefits of emapalumab for patients with CRS, it is also necessary to admit the limitations of the study. First, this single-center, retrospective, small-sample study is subject to potential bias and limited generalizability, and it also impairs the ability to detect rare yet severe AEs; Second, the follow-up duration of this study is sufficient to assess the acute control efficacy of emapalumab for CRS, but it fails to evaluate its impact on the long-term survival of patients. Future prospective studies with larger samples and longer follow-up periods are required to comprehensively assess its long-term benefits and risks. Finally, this study lacks a control group. However, currently, designing a control group of “emapalumab-only treatment without prior first-line agents” was limited by clinical practice.

## Conclusion

Our findings preliminarily confirmed that emapalumab is an effective salvage therapy for CAR-T-associated CRS patients refractory to low-dose glucocorticoids and/or tocilizumab. It rapidly alleviates CRS-related symptoms, achieves remission, and seems to exert no observable adverse effects on the anti-tumor activity of CAR-T cells.

## Data Availability

The original contributions presented in the study are included in the article/supplementary material. Further inquiries can be directed to the corresponding authors.
